# Use of belimumab in real-world in Spain: a scoping review about characteristics of SLE patients

**DOI:** 10.1007/s10067-022-06287-9

**Published:** 2022-07-23

**Authors:** Carlos Rodríguez Escalera, Ángela María Zurita Guisado, Francisco Javier Mateo, Noemí Bahamontes-Rosa, María Jesús García Villanueva

**Affiliations:** 1 Rheumatology department, Hospital Marítimo de Torremolinos, Málaga, Spain; 2 Medical department, GSK, P.T.M Severo Ochoa, 2 - 28760. Tres Cantos, Madrid, Spain; 3 Rheumatology department, Hospital Universitario Ramón y Cajal, Madrid, Spain

**Keywords:** B-cell-targeted therapy, Belimumab, Real-life experience, Real-world data, Steroid sparing, Systemic lupus erythematosus

## Abstract

**Background:**

Belimumab was the first biological drug approved for Systemic Lupus Erythematosus (SLE). There is not a review focusing on all real-life experience with belimumab to date that could help to describe how this drug behaves in the Spanish clinical setting.

**Objective:**

To describe the characteristics of SLE patients treated with belimumab added to standard of care in real-clinical setting in Spain.

**Methods:**

We conducted a comprehensive scoping review of real-world data (RWD) according to PRISMA Scoping Reviews Checklist and the framework proposed by Arksey and O’Malley. PubMed and EMBASE were searched without language restriction and hand searches of relevant articles were examined.

**Results:**

We included data from 222 patients treated with belimumab for SLE included in 19 RWD studies conducted in Spain. The mean age was 40.9 years, 84.2% were female, and baseline scores SELENA-SLEDAI ranged between 5.9 and 12. Lupus nephritis basal prevalence was of 2.7%. The main reason for belimumab initiation was previous treatments lack of efficacy (69.7%) and the most common laboratory abnormalities were hypocomplementemia (40.9%), ANA + (34.2%), and anti-DNA (33.3%). The addition of belimumab to standard therapy was associated with a reduction of daily glucocorticoids intake in 1.4–11.1 mg at 6 months. Belimumab discontinuation was observed in 18.6% of patients.

**Conclusion:**

Our study helps to further explore the profile of SLE patients most likely to be treated with belimumab.

**Supplementary Information:**

The online version contains supplementary material available at 10.1007/s10067-022-06287-9.

**Table:** 

**Key Points** • *Scientific evidence in SLE provided by randomized controlled trials sometimes differs from the actual treatment of SLE patients in routine clinical practice.* • *There is a lack of published “real-world” data on SLE treatment with belimumab in Spain.* • *This scoping review intends to describe and analyze the clinical characteristics of SLE patients receiving belimumab in a real-life setting in Spain.* • *These “real-world” clinical experience can provide a more realistic view of the overall patterns of SLE care compared with clinical trials.*	

## Introduction

Systemic lupus erythematosus (SLE) is a chronic autoimmune disease with a heterogeneous presentation, caused by a combination of factors, being a prominent feature the production of high levels of antinuclear antibodies (ANAs) [1]. Incidence rates in Spain are 2–3 cases/100,000 inhabitants per year [2, 3]. Importantly, patients with SLE are at risk for significant morbidity [4] and mortality [5, 6].

SLE is associated with an unpredictable course and most patients require lifelong medication. SLE pharmacological approach is heterogeneous [7]. However, prolonged treatment is associated with significant morbidity and adverse side effects, especially high-dose corticosteroids and immunomodulatory therapies [8, 9]. To allow the reduction of such drugs toxicity, efforts are being made to develop drugs with a more selective mechanism of action such as biological therapies. One of the numerous immune defects in patients with SLE is an increased circulating level of a B-cell survival factor (known as BLyS or B cell-activating factor -BAFF-). Belimumab is a human recombinant immunoglobulin G 1λ (IgG1λ) monoclonal antibody that selectively binds the soluble form of BLyS to its receptors on B cells neutralizing its biological activity. Thus, belimumab inhibits the survival of B cells and reduces the differentiation of B cells into immunoglobulin-producing plasma cells [10]. In 2011, belimumab was approved by European Medicines Agency (EMA) as add-on therapy in adult patients with active SLE, becoming the first and only biological drug approved for SLE treatment [11]. Furthermore, belimumab has proved its efficacy and safety in patients with pediatric SLE and, more recently, in adults with active lupus nephritis gaining both approvals for indication by the EMA [10]. More recently, positive results of a phase III trial with belimumab in patients with active lupus nephritis (LN), a significant cause of morbimortality SLE patients, have been published [12, 13]. Despite the encouraging scientific evidence provided by randomized controlled trials (RCTs) [14–16], treatment of SLE patients in routine clinical practice differs from that established on the controlled setting of clinical trials and physicians can find themselves in the difficult situation of using biological drugs as off-label agents when trying to control a refractory disease [17]. These “real-world” clinical experience from practice settings can provide a more realistic view of the overall patterns of SLE care. Real-world evidence includes analysis of real-world data (RWD) gathered from non-conventional sources, including patient registries, observational studies, and social media, among others [18]. In Spain, RWD on belimumab clinical benefit in everyday clinical practice are available and it has been independently published as case reports, case series, and observational studies; however, to our knowledge, there is not a review focusing on all real-life experience with belimumab in SLE patients in Spain to date that could help to describe how this drug behaves in the routine clinical setting. Therefore, we conducted a literature review that may shed some light on real-life use of belimumab.

## Methods

The PRISMA extension for Scoping Reviews (PRISMA-ScR) [19] Checklist and the framework proposed by Arksey and O’Malley [20] were used to guide this review. The research question was: what are the clinical characteristics of SLE patients receiving belimumab in clinical practice in Spain? The following steps were followed:

### Search strategy

An electronic literature search was conducted in the two major databases (PubMed and EMBASE) for the identification of relevant real-world publications on the use of belimumab in SLE patients in clinical practice in Spain. Articles published between database inception to 19th November 2019 were included in the review. The following main search terms were used based on inclusion and exclusion criteria: “Belimumab”, “lupus”, “observational study”, “pragmatic trial”, “practical trial”, “case–control studies”, “cohort studies” and “real-world evidence”. Most of the articles were found using combinations between Boolean operators “AND/OR”, search terms and synonyms for the keywords. The remaining articles were found using a hand search of reference lists of original studies included in the review after searching the two databases. The search was restricted to studies in humans, but no language or time restrictions were imposed.

### Study selection

Following removal of duplicates, the titles and abstracts of retrieved articles were screened and reviewed the full texts. Full texts of retrieved publications were reviewed and marked for inclusion if they (1) included SLE patients treated with belimumab, (2) were conducted in clinical practice in Spain, and (3) presented real-world evidence. Papers were excluded if they did not fit into the conceptual framework of the study. The quality appraisal was not performed in accordance with the standard approach to conducting scoping reviews [20, 21].

### Charting data and reporting the results

A data extraction template was developed to determine which variables to extract for each study. All data were entered and verified onto a “data charting form” continuously updated in an iterative process using the database program Excel. The following data were extracted from full text publications selected for inclusion in the review: author(s), year of publication, study location, title of the publication, intervention type and duration, study population (i.e., mean age, mean SLE duration, main manifestations, concomitant treatments), study methodology (i.e., case report, retrospective or prospective case series), outcome measures, and important results of the publication on the effectiveness and safety of belimumab. We grouped the results according to the study design and summarized the type of settings and populations characteristics for each group, along with the outcomes used and broad findings.

## Results

**Fig. 1:**
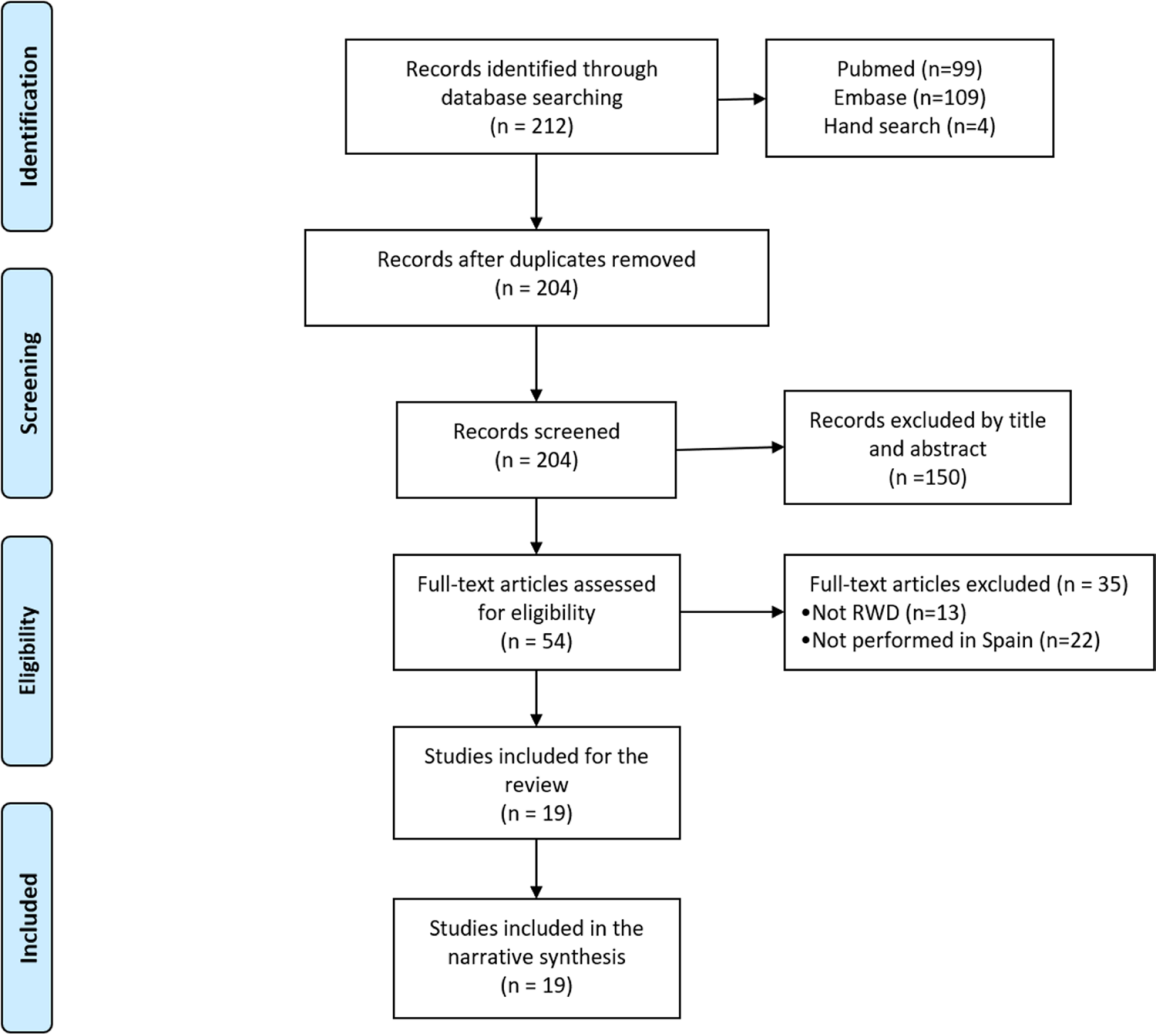
PRISMA flowchart with the main stages of the review process

From a total of 212 real-world publications identified, 19 Spanish publications were finally included. Two congress abstracts [22, 23] were based on the same dataset that 2 included articles [24, 25] but focused on different aspects of the dataset and so were both included in this review. The PRISMA flowchart of this process is provided in Fig. 1.

### Characteristics of included studies

The 19 studies selected in the current review were published between 2014 and 2019; with the exception of 6 descriptive case reports [26–32], all publications were case series: 3 prospective [33–35] and 10 retrospectives [23–25, 32, 36–41]. Three studies were conducted in hospital settings from Madrid [34, 38, 40], 3 in Barcelona [26, 29, 39], 2 conducted in Murcia Region [31, 41], and one each other in Alcalá de Henares [35], Alicante [33], Ciudad Real [28], Granada [30], Pamplona [36], Torrelavega [37], Valencia [24], and Vizcaya [27]. The remaining 3 publications [22, 32, 42] were multicenter studies. The number of included patients varied widely within these case series from 5 [40] to 64 [22] SLE patients.

### Baseline characteristics of patients receiving belimumab

A total of 222 patients were treated with intravenous belimumab 10 mg/Kg and one case report [26] described the first switch to subcutaneous belimumab 200 mg/week. The mean age was 40.9 years (from 25 to 56 years) and 187 patients (84.2%) were female. Reasons for belimumab initiation in retrospective case series and case reports, when data were available, included ineffective previous treatment, reported for *n* = 67 patients (69.7%); refractory SLE articular manifestations (*n* = 24, 25.0%); and refractory SLE mucocutaneous manifestations (*n* = 5, 5.2%).

Only 3 retrospective analysis reported the baseline average activity score (SELENA-SLEDAI), with scores ranging from 5.9 [35] to 12 [42] (mean 9.1). In general terms, the mean duration of disease from onset of symptoms to diagnosis ranged between 10 months [31] and 27 years [33] (mean duration 9.0 years), with a shorter duration for case reports subgroups. Baseline clinical characteristics of patients according to the 3 design study groups are shown in Table 1.

**Table 1:** Clinical characteristics of SLE patients according to the 3 design study groups

	Patients, *n*	Male (female)	Mean age, years (min–max)	Mean SLE duration, years (min–max)	Mean SELENA-SLEDAI score (min–max)
Case reports^26–31^	6	0 (4) + 2 NR	43 (25–56)	5.8 (0.8–14)	8 (8–8)*
Retrospective Studies^23–25,32,36–41^	193	18 (175)	39.3 (28–48.5)	10.7 (8.0–14)	10.3 (9.5–12)
Prospective Studies^33–35^	23	0 (8) + 15 NR	46 (25–65)	18 (7–27)	6.7 (5.9–7.6)

*NR* not reported. *Data from only one case report

All patients included presented at least one clinical manifestation of SLE. Considering all studies, the most frequently reported manifestations were articular (mainly arthritis) (*n* = 95, 42.8%) and mucocutaneous (*n* = 75, 33.8%). Hematological co-morbidities (*n* = 29, 13.1%), renal involvement (*n* = 16, 7.2%), lung or heart involvement (*n* = 11, 5.0%), and vasculitis (*n* = 4, 1.8%) were documented. In two retrospective analysis [36, 39] and one case report [26], 7 patients were reported to have antiphospholipid syndrome and other 3 retrospective analyses [39, 40, 42] included 4 patients with LN. Only 3 patients were reported as associated Sjögren syndrome and 2 as autoimmune thyroiditis [39].

Regarding the serological profile, hypocomplementemia (low C3/C4), ANA + , and anti-DNA were the most common in the analysis of laboratory abnormalities with 40.9%, 34.2%, and 33,3% of patients, respectively. Autoantibody anti-Ro was only reported in 7.1% of patients, and anti-La and anti-SM were positive in 4.06% and 3.5%, respectively. Prevalence of other autoantibodies such as antiphospholipid, anti-SM, anti-RNP, anti-SSA, anti-SSB, and anti-Smith was positive in less than 5% of patients.

### Previous treatments to belimumab

Only 5 retrospective studies [32, 36, 37, 39, 42] and 6 case reports [26–31] (*n* = 93 patients) provided data about the number and type of previous treatments to belimumab in included patients. None of the prospective studies described any information about this outcome. No data about the duration of each treatment were reported, although one retrospective study [42] registered a mean of 7 years of previous treatment.

Considering a total of 93 patients for which data was available, this review shows that 61 patients (65.5%) were under antimalarial treatment before starting with belimumab and 38 (40.8%) were treated with corticosteroids. Hydroxychloroquine was the most common used treatment for patients treated with antimalarials (*n* = 39, 41.9%). Regarding corticoids, prednisone was the most prescribed drug previously to belimumab (*n* = 25, 26.8%) followed by others less used such as prednisolone and clobetasol (each one *n* = 1, 1.08%). Seventy-one patients (76.3%) had received at least one immunosuppressive agent before starting treatment with belimumab. In fact, one retrospective analysis [39] showed that the number of previous immunosuppressants was 2.2 ± 1.1. Azathioprine (*n* = 47, 50.4%), methotrexate (*n* = 39, 41.9%), and mycophenolate (*n* = 33, 35.4%) were the immunosuppressants most commonly prescribed, while less than 25% of patients had received treatment with others drugs such as leflunomide, cyclosporine, tacrolimus, or pimecrolimus. Other SLE-related medications received previously to belimumab included rituximab (*n* = 12, 12.9%) and cyclophosphamide (*n* = 11, 11.8%) followed by anti-TNF (*n* = 3, 3.2%), thalidomide (*n* = 2, 2.1%), efalizumab (*n* = 1, 1.08%), abatacept (*n* = 2, 2.1%), etanercept (*n* = 1, 1.08%), and adalimumab (*n* = 1, 1.08%).

### Concomitant treatment to the infusion of belimumab

Data about treatments concomitant to belimumab were provided by 6 retrospective analysis [24, 25, 36, 37, 39, 42], 1 prospective study [33] and 5 case reports [26, 27, 29–31] (*n* = 158). Data on the overall use of corticosteroids were available for 140 patients (88.6%) with oral prednisone as the most frequently used corticosteroid in combination with belimumab treatment (*n* = 44, 27.8%). When reported, doses of prednisone varied widely within studies ranging from 5 to 30 mg/day and one retrospective analysis [39] reported a mean dose of prednisone of 10.2 ± 1.8 mg/day. Only one case report [31] notified the concomitant use of 500 mg of methylprednisolone in 3 doses.

In addition to corticosteroids, a proportion of patients of the total for which data was available were receiving antimalarials (*n* = 52, 32.9%) and immunosuppressants (*n* = 61, 38.6%) while injectable/intravenous belimumab was administered. Between antimalarials, 46 patients (29.1%) received hydroxychloroquine; however, insufficient information was provided about mean doses used. Only one case report [30] notified the use of 200 mg daily of hydroxychloroquine. Regarding immunosuppressants, mycophenolate (*n* = 22, 13.9%) and methotrexate (*n* = 20, 12.6%) were the most used followed by azathioprine (*n* = 17, 10.7%) and leflunomide (*n* = 3, 1.9%), and tacrolimus (*n* = 1, 0.6%) with less use or residual.

### Belimumab: overall effects

Data from prospective studies [33–35] showed that addition of belimumab to usual treatment in SLE patients was associated with (1) decreased disease activity measured by the Systemic Lupus Erythematosus Disease Activity Index (SLEDAI) score (up to 70%) and (2) lower absolute CD19 + B cell count and/or anti-DNA antibody levels. In retrospective studies [24, 25, 36–42] and case reports [26–31], belimumab also reported clinical benefits improving overall symptoms. Only one retrospective study [40] provided data about belimumab effects on SLE flares, in which the patients treated with belimumab revealed sustained improvement in cutaneous manifestations than that which they had under standard treatment. In addition, in a multicenter study developed by Cortés et al. [25], the use of belimumab was associated with a reduction of the use of health resources (emergency-room visits or unscheduled visits to treating-physician, between other outcomes). Belimumab was generally well-tolerated; most common registered adverse events were hematological disorders, infections, and asthenia.

### Effects of treatment with belimumab on use of corticoids

Data about the reduction of daily corticoids intake after starting belimumab treatment in SLE patients were only provided by 7 retrospective studies [24, 25, 32, 36, 38, 39, 41] (*n* = 166) and 3 case reports [26, 27, 29]. None of the included prospective studies provided useful data about this outcome. Retrospective studies showed that addition of belimumab to standard therapy was associated with a reduction of daily glucocorticoids intake in mean doses ranging from 1.4 to 11.1 mg at 6 months after treatment with belimumab [25, 32, 36, 39] (*n* = 130) and in a 50% reduction after 36 weeks [41] (*n* = 5). One retrospective study [39] reported corticosteroids mean dose reduction of 3.8 mg/day at 12 months post-treatment with belimumab for 23 patients. In the same study [39] and during the 24 months post-treatment, 23 patients experienced a mean dose reduction of 5.6 mg/day. In other retrospective study [24], lower corticosteroids dose reductions were observed after 29 months of treatment with belimumab with mean values of 4.02 mg/day (*n* = 19). Corticoids (prednisone) could be discontinued in two patients, and in only four the dose was greater than 7.5 mg/day [39]. Only one retrospective study [36] registered increases in 0.6 mg/day mean corticosteroids dose, both at 6 months (*n* = 16) and at 12 months (*n* = 7) after treatment. The three case reports and one retrospective study [32] also reported that belimumab acted as a corticosteroid-sparing agent with the ability to reduce corticosteroid daily intake, but only one [26] quantified the reduction of 15 mg/day at 7 months while in other study [32], belimumab allowed to reduce the corticosteroids by 68% during the first year and achieved to a low dose (< 7.5 mg/day) in two patients with severe renal involvement (> 1 g proteinuria/24 h).

### Effects of discontinuation of treatment with belimumab

Only 9 retrospective studies [24, 25, 32, 36–41] provided data about the discontinuation of belimumab and/or reasons for discontinuation. One case report [29] notified that belimumab was stopped due to the patient achieving complete remission. Belimumab discontinuation was observed in 36 of 193 patients (18.6%) during a mean follow-up of 18.5 ± 17.4 months. Most common cause of discontinuation reported was lack of efficacy/response (*n* = 13, 36.1%), followed by more specific reasons such as persistence of arthritis (*n* = 4, 11.1%), worsening of cutaneous involvement and oral ulcers (6 months after starting belimumab) (*n* = 2, 5.5%), neutropenia (*n* = 2, 5.5%), or lupus nephritis (12 months after starting belimumab) (*n* = 2, 5.5%). Other less common reasons were thrombosis, maintenance of pleural effusion (6 months after starting belimumab), urothelial carcinoma, pelvic inflammatory disease (within the first 6 months of therapy), peripheral venous insufficiency, pregnancy, itchy skin lesions, uncertain drug allergy, worsening proteinuria, and primary pulmonary hypertension (each one *n* = 1, 3.5%). Number and detailed reasons for discontinuation of belimumab treatment are reported in Table 2.

**Table 2:** Number and detailed reasons for discontinuation of belimumab treatment

Author, year	Study design	*n*	Reasons
Carrion-Barbera, 2019	CR	0	
Castillo Dayer, 2019	CR	0	
Gimenez, 2019	CR	0	
Gonzalez-Echavarri, 2016	CR	0	
Husein-El Ahmed, 2014	CR	0	
Carbajal, 2017	CR	1	NR
Hernandez-Florez, 2015	PS	0	
Lorente, 2018	PS	0	
Montserrat, 2016	PS	0	
Aldasoro, 2018	RS	4	Lack of efficacy (persistence of arthritis)
Almanchel, 2014	RS	1	Thrombosis
Alonso, 2014	RS	3	Lack of response (2) Prolonged adverse reactions (neutropenia) (1)
Anjo, 2019	RS	4	Inadequate response (6 months after starting belimumab) with worsening of cutaneous involvement and oral ulcers and maintenance of pleural effusion (*n* = 3) Development of lupus nephritis class IV (12 months after starting belimumab) (*n* = 1)
Argumanez, 2019	RS	4	Ineffectiveness (*n* = 2) Adverse events: neutropenia and urothelial carcinoma (*n* = 2)
Cortes, 2014	RS	2	Lack of efficacy (*n* = 1), Pelvic inflammatory disease (*n* = 1)
Navarro, 2019	RS	5	Peripheral venous insufficiency (*n* = 1) Pregnancy (*n* = 1) Itchy skin lesions (*n* = 1) Uncertain drug allergy (*n* = 1) Primary pulmonary hypertension (*n* = 1)
Riancho-Zarrabeitia, 2018	RS	4	Inefficacy (*n* = 3) Lupus nephritis class IV (*n* = 1)
Moriano, 2018	RS	8	Inefficacy (*n* = 5) Pregnancy (*n* = 1) Worsening proteinuria (*n* = 1)
Brito-Zeron, 2014	RS	NR	NR

*NR*, not reported; *CR*, case report; *RS*, retrospective studies; *PS*, prospective studies

The effects of belimumab discontinuation were analyzed only by one case report [29] in which, 4 months after the discontinuation of belimumab, the patient suffered a severe SLE flare consisting of a new class IV LN and severe pericardial effusion with myocarditis. The patient received treatment with glucocorticoids and intravenous cyclophosphamide pulses but, owing to progressive worsening of renal function and pericardial effusion, combined treatment of rituximab, intravenous immunoglobulins, and plasma exchange was administered, along with renal replacement therapy (RRT). One month after this treatment, pericardial effusion resolved, with normalization of the ventricular ejection fraction and immunological activity. However, after 6 cyclophosphamide boluses (cumulated dose of 3 g) and 4 months of follow-up, the patient still needed RRT.

### Lupus nephritis: prevalence and management

Five included studies provided data about LN prevalence at baseline or during follow-up: 3 retrospective analyses [39, 40, 42] and 2 case reports [27, 29]. LN was presented in a total of 6 patients (2.7%), all in class IV (when this data was available). In addition, one retrospective analysis [24] included 7 patients with renal clinical manifestations at the onset of study and only 3 at the end, but insufficient information was provided about whether renal manifestations were LN or another type of manifestation.

Regarding LN management, only 2 case reports provided detailed information [27, 29]. In one of them, one patient showed a renal biopsy of LN class IV and received treatment with high-dose prednisone and cyclophosphamide and a number of different therapies, including four cycles of rituximab. After 1 year in complete remission, the patient presented again with edema and hypertension, and proteinuria increased after therapy with methylprednisolone pulses, immunoglobulin, or cyclophosphamide. Belimumab (10 mg/kg at weeks 0–2–4, then every 4 weeks) was added to treatment and proteinuria started to decrease at month 2, and 4 months after the first infusion of belimumab, complete remission was achieved. As described in the previous section, in the other case report [29], one patient suffered a new severe SLE flare consisting of a new class IV LN and severe pericardial effusion with myocarditis 4 months after the discontinuation of belimumab.

In retrospective studies, one analysis [40] reported 2 patients with LN at the onset of study. In this study, 3 patients had to discontinue belimumab treatment, but detailed information about if these patients had renal involvement was not provided. In other study [42], one patient with refractory LN was included at the onset and, although after a mean follow-up of 7 months, 8 patients were classified as responders (improvement greater than 80% in 5 patients), authors did not provide detailed information about if patient with refractory LN was one of them. At last, in other retrospective case series [39], belimumab was withdrawn after 12 months of treatment after starting belimumab due to development of class IV LN.

## Discussion

This scoping review describes the clinical characteristics of SLE patients receiving belimumab in real-life settings in Spain. The included case series and case reports conducted in different Spanish sites provide RWD related to the SLE patient profile in treatment with belimumab allowing to know medication treatment patterns, use of corticoids, effects of discontinuation of belimumab treatment, and effects on specific populations (i.e., LN patients). According to EULAR recommendation about standard-of-care based on combinations of hydroxychloroquine and corticosteroids with or without immunosuppressive agents [43], oral prednisone (with a range of doses 5–30 mg/day) was the corticosteroid most frequently used as concomitant treatment with belimumab (27.8%), followed by antimalarials (mainly hydroxychloroquine) and immunosuppressants (mainly mycophenolate and methotrexate). The addition of belimumab to standard treatment in SLE patients was associated with decreased disease activity measured by SLEDAI score (up to 70%), lower absolute CD19 + B cell count and/or anti-DNA antibody levels, and clinical benefits improving overall symptoms. The addition of belimumab to standard therapy was also associated with a reduction of daily glucocorticoids mean intake in 1.4–11.1 mg at 6 months and in a 5.6 mg/day at 24 months, and even, corticoids could be discontinued in two patients. Due to a significant proportion of the organ damage in SLE patients that could be attributed to corticosteroid therapy (glucocorticoid-induced avascular necrosis of the hips and knees, osteoporosis, fatigue, and cognitive dysfunction, particularly) [44], this steroid-sparing effect associated to belimumab may reduce organ damage progression [45]. Belimumab discontinuation was observed in only 18.6% of patients during a mean follow-up of 18.5 months, mainly, due to lack of efficacy/response to treatment. In one patient, belimumab discontinuation led to a severe SLE flare after 4 months (class IV LN and severe pericardial effusion with myocarditis). LN prevalence was of 2.7%, all in class IV, and off-label therapy with belimumab in one patient led to complete remission at 4 months after the first infusion.

Characteristics of SLE patients on treatment with belimumab have been also studied in patient populations from clinical trials [16, 46] and from a study in 24 Italian centers [47]. The included patient population in Spain real-world setting was similar to those seen in clinical trials on belimumab. Similar values were found between our populations and clinical trials [16, 46, 47] relative to mean duration of SLE (9.0 vs 8 years, respectively) and baseline SELENA-SLEDAI activity score (9.1 vs 9.6 points). In our real-world population, as in clinical trials, the most common laboratory abnormalities were hypocomplementemia, ANA + , and anti-DNA, although in the present study, the prevalence was lower than observed in clinical trials (34.2% vs 72% for ANA + and 33.3% vs 50% for anti-DNA, respectively) and higher than observed in the Italian multicenter study [47] (33.3% vs 28.6% for anti-DNA).

Relevant differences were found regarding baseline medication use pattern while comparing both populations. In the BLISS-52 [16] and BLISS-76 trials [46], 68% of patients used daily prednisone, 70% antimalarials, and 50% immunosuppressants in comparison to 40.8%, 65.5%, and 76.3% registered in the present study. Differences in those findings could be due to missing data included in the RWD studies analyzed (for example, only 6 studies [26, 27, 30, 31, 36, 39] provided data about prednisone use).

In view of our findings, we observed a shift on the use of belimumab in SLE patients. In earlier studies (published in 2014), belimumab was most commonly prescribed for the treatment of musculoskeletal (mainly articular) manifestations, while in more recent studies (published in 2018), the mucocutaneous manifestations were the most common indications. Due to missing data in included studies, other trends in the use of belimumab could not be analyzed.

One of the strengths of this study was the analyses of the effects of belimumab discontinuation. We identified one case of rebound phenomenon after treatment with belimumab where the patient suffered a severe SLE flare 4 months after the discontinuation. Similar results were seen in other studies suggesting a possible rebound effect following belimumab withdrawal that could be due to an increase in BAFF levels and led to a disease flare [48]. However, other studies have suggested that a temporary (24-week) discontinuation of belimumab in patients with low disease activity do not appear to increase the risk of SLE flares or rebound across 52 weeks of follow-up [49]. Another key finding of this study was the reduction of corticosteroids dosage observed, supporting the steroid-sparing effect of belimumab also observed in post hoc analyses of belimumab clinical trials and the long-term extension study [50, 51].

In a multicenter study developed by Cortés et al. [25], OBSErve study, the use of belimumab was associated with a reduction of the use of health resources (emergency-room visits or unscheduled visits to treating-physician, between other outcomes). These findings are supported by the study developed by Cevey M et al. [52] in 2020.

This is, up to our knowledge, the first scoping review describing the different clinical characteristics of SLE patients treated with belimumab in Spain. The study has some limitations that could lead to some risk of bias. Firstly, the analyses were conducted with relatively low patient numbers (*n* = 222), reflecting the lack of scientific evidence about characteristics of SLE patients in Spain treated with belimumab; secondly, another limitation is the lack of information observed regarding background therapy; and at last, missing data about the period of observation and short follow-up in some of the included studies may be inadequate for patients with a clinical history of disease flares followed by long periods of remission.

In terms of patient population, there is a lack of patients with pediatric SLE and a small number of LN patients included because of the limitation of available literature; given the recent EMA approval for the therapeutic indication of belimumab in patients with LN, and the low prevalence of childhood SLE (cSLE), no cases have been yet published of belimumab in LN and cSLE in Spain [10].

Regarding how the COVID-19 pandemic has affected the use of belimumab, there is not evidence yet on the use of this drug for these patients. However, some studies about the immunological response to the vaccine against COVID-19 in patients treated with belimumab have been published indicating the retention of immunogenicity of COVID-19 vaccine [53].

Notwithstanding the limitations mentioned above, we think that our findings may help to better define the profile of SLE patients who may obtain clinical benefit with belimumab treatment, thereby demonstrating the usefulness for the design of future belimumab treatment strategies for SLE patients in Spain.

## Conclusions

RWD from studies conducted in different Spanish sites show that the most common characteristics of SLE patients treated with belimumab are female, around 40 years of age, with baseline scores SELENA-SLEDAI between 5.9 and 12 and a wide range of mean duration of disease. According to EULAR recommendations about considering add-on treatment with belimumab in patients with inadequate response to standard-of-care [43], the main reason for belimumab initiation is ineffective previous treatments and the most frequently reported SLE manifestations are articular. The addition of belimumab to standard therapy is associated with a reduction of daily corticosteroids intake and, potentially, prevention of organ damage accrual. Belimumab discontinuation is observed in 18.6% of patients. Our findings, in line with results from clinical trials, may bring some clarity on real-life use of belimumab and help to design potential future treatment strategies with belimumab for patients with SLE in Spain.

## Supplementary Information

Below is the link to the electronic supplementary material.

Supplementary file1 (DOCX 45 KB)
