# Supplementary material for: Use of belimumab in real-world in Spain: a scoping review about characteristics of SLE patients
Source: Clin Rheumatol. 2022 Jul 23;41(11):3373–82. doi: 10.1007/s10067-022-06287-9 (PMC9308486; doi:10.1007/s10067-022-06287-9)
Supplement: Supplementary file 1 — Supplementary file1 (DOCX 45 KB) [file 10067_2022_6287_MOESM1_ESM.docx]

Supplementary material

| TABLE 1. MAIN DEMOGRAPHIC CHARACTERISTICS, CLINICAL MANIFESTATIONS, AND INTERVENTIONS OF INCLUDED PATIENTS: CASE REPORTS | | | | | | | | |
| --- | --- | --- | --- | --- | --- | --- | --- | --- |
| *Author, year* | *Number of patients (n)* | *Male (Female)* | *Mean age, years (range)* | *Diagnosis* | *Mean SLE duration, years (range)* | *Mean basal SLEDAI score (E-L) (±SD)* | *Concomitant treatments, n (%)* | *Intervention* |
| *Carrión-Barberà, 2019* | *1* | *0 (1)* | *51* | *SLE Rheumatoid arthritis, antiphospholipid antibodies +* | *2* | *8* | *Hydroxychloroquine 400 mg/12 h combined with leflunomide 20 mg / day and with low-dose prednisone erratic control, justifying change of immunosuppressive therapy to methotrexate 15 mg / week. Aspirin 100 mg / day* | *Intravenous Belimumab was started at a dose of 10 mg/kg/month as a corticosteroid-sparing agent 1 dose of subcutaneous Belimumab after 24 doses of Belimumab* |
| *Castillo-Dayer, 2019* | *1* | *0 (1)* | *31* | *SLE Polyarticular, nodular, and erosive rheumatoid arthritis Rheumatoid factor and anticyclic citrullinated peptide +* | *9* | *NR* | *500 mg of methylprednisolone in 3 doses, hydroxychloroquine dose was increased to 400 mg daily* | *Intravenous Belimumab (560 mg) monthly* |
| *Giménez, 2019* | *1* | *0 (1)* | *56* | *SLE* | *NR* | *NR* | *Prednisolone to 1 mg / kg / day with gradual reduction* | *Belimumab at a dose of 10 mg/kg in days 0, 14 and 28 and subsequently at intervals every 4 weeks* |
| *Gonzalez-Echavarri, 2016* | *1* | *NR* | *25* | *SLE based on arthritis, fever vasculitic skin lesions, antinuclear antibody +(1: 600), anti-dsDNA antibody (1: 200) and hypocomplementemia (C3: 53 mg / dL, C4: 3 mg / dL).* | *14* | *NR* | *Hydroxychloroquine, prednisone 5 mg / day, mycophenolate (750 mg / day), tacrolimus (7 mg / day), enalapril* | *Belimumab (10 mg / kg) in 0-2-4 weeks, then every 4 weeks* |
| *Husein-ElAhmed, 2014* | *1* | *0 (1)* | *52* | *SLE with kidney and musculo‑skeletal involvement Antinuclear antibody (1/640) and anti-Ro / SSA +* | *6* | *NR* | *Maintenance therapy with Hydroxychloroquine 200 mg daily and prednisone 20 mg daily* | *Belimumab at a dose of 10 mg / kg / day on days 1, 14 and 28.* |
| *Carbajal, 2017* | *1* | *NR* | *NR* | *SLE based on malar rash, arthritis, anti- nuclear antibodies +(ANAs), anti-double-stranded DNA antibodies (anti-dsDNA), hypocomplementaemia,*  *haemolytic anaemia, and biopsy-proven class IV lupus nephritis* | *NR* | *NR* | *Glucocorticoids, hydroxychloroquine and mycophenolate mofetil Induction therapy with glucocorticoids and cyclophosphamide outbreak, followed after remission maintenance therapy with azathioprine* | *Treatment with Belimumab was added to glucocorticoids, hydroxychloroquine and Mycophenolate Belimumab suspension after 14 months of treatment* |
| *SLE: Systemic Lupus Erythematosus; SLICC: Systemic Lupus International Collaborating Clinics; SLEDAI: Systemic lupus erythematosus disease activity index; NR: Not reported; IQR: Interquartile range* | | | | | | | | |

| TABLE 2. MAIN DEMOGRAPHIC CHARACTERISTICS, CLINICAL MANIFESTATIONS, AND INTERVENTIONS OF INCLUDED PATIENTS: RETROSPECTIVE STUDIES | | | | | | | | |
| --- | --- | --- | --- | --- | --- | --- | --- | --- |
| *Author, year* | *Number of patients (n)* | *Male (Female)* | *Mean age, years (range)* | *Diagnosis* | *Mean SLE duration, years (range)* | *Mean basal SLEDAI score (E-L) (±SD)* | *Concomitant treatments, n (%)* | *Intervention* |
| *Aldasoro, 2018* | *18* | *2 (16)* | *34.3*  *(IQR 27-45.7)* | *SLE with antiphospholipid autoantibody + (n = 13) ANA + (n = 17) Anti-Ro + (n = 9) Anti-nRP (n = 4) Anti-SM (n = 4) Anti-La (n = 4)* | *8,05*  *(3.54 to 14.02)* | *10.00 (8 to 13.7)* | *Prednisone (n = 17) Hydroxychloroquine (n = 13), Methotrexate (n = 7),  Azathioprine (n = 1), Mycophenolate (n = 1) Leflunomide (n = 1)* | *Belimumab bolus with a median of 8.5*  *(IQR 2-32.7)* |
| *Almanchel, 2014* | *5* | *0 (5)* | *36 (25-50)* | *SLE with ANA +, anti-DNA +, Low C3 and C4* | *NR* | *NR* | *Prednisone* | *Treatment with Belimumab* |
| *Alonso, 2014* | *6* | *0 (6)* | *38* | *SLE with articular involvement (n = 6),*  *cutaneous involvement (n = 5),*  *haematological involvement (n = 2) and*  *renal involvement (n = 2)* | *NR* | *NR* | *Alone or in combination: corticosteroids (Prednisone), antimalarials (Hydroxychloroquine) or immunosuppressants (Mycophenolate mofetil)* | *Belimumab standard dose 10 mg / kg* |
| *Anjo, 2019* | *23* | *0 (23)* | *41.5 ± 10.5* | *SLE (n = 20) Cutaneous discoid lupus (n = 2) Obstetric anti-phospholipid syndrome (n = 1) Sjögren’s syndrome-associated (n = 3) Anti-phospholipid syndrome-associated (n = 2) Autoimmune thyroiditis (n = 2)* | *171.8 (months) ± 131.1* | *9.6 ± 1.6 (SLEDAI-2K score)* | *Hydroxychloroquine (n = 22),*  *Prednisone (n = 23),*  *immunosuppressants (n = 22),*  *Azathioprine (n = 6), Mycophenolic acid (n = 8), Methotrexate (n = 6), Cyclosporine (n = 0),  Leflunomide (n = 2)* | *Belimumab with a duration of 18.9 months*  *(± 16.0)* |
| *Argumanez, 2019* | *12* | *0 (12)* | *48.5 (31-70)* | *SLE with arthritis (n = 7),*  *thrombocytopenia (n = 3),*  *cutaneous (n = 2),*  *serositis (n = 1)* | *NR* | *NR* | *NR* | *Belimumab with a duration of 27.5 months (+/- 26.24)* |
| *Cortes, 2014* | *64* | *7 (57)* | *42.7 ± 12* | *SLE with musculoskeletal manifestations (arthritis = 56.2%), immunologic (low C3, C4 or CH50 = 53.1%), Increased anti-dsDNA antibody levels (48.44%) mucocutaneous (rash 26.56%).* | *23% were diagnosed with SLE <5 years* | *10.1* | *Oral steroids (95%)* | *Belimumab (10mg/kg) with an average duration of 6 months* |
| *Brito-Zeron, 2014* | *10* | *1 (9)* | *41.8 (24-71)* | *SLE with mucocutaneous involvement (n=4), refractory vasculitis (n=3),*  *systemic disease (n=2),*  *refractory lupus nephritis (n=1),*  *high titers of anti-dsDNA and/or hypocomplementemia (n = 8)* | *NR* | *12 (6-33)* | *Corticosteroids (n = 10), antimalarials (n = 6) immunosuppressive agents (n = 8) (mycophenolate n = 6)* | *Belimumab (10 mg / kg)* |
| *Moriano, 2018* | *25* | *7(18)* | *43,7 ±12,2* | *SLE with arthritis n=11,*  *mucocutaneous (n=5)*  *immunologic findings (n=5)*  *severe renal involvement (>1 g proteinuria/24h) (n=2)* | *9,5 ±7,1* | *9.5* | *NR* | *Belimumab in actual clinical practice* |
| *Navarro, 2019* | *15 (Sept 2017) 19 (Dec 2018)* | *1 (18)* | *32 (8.34)  12 28 (14 Q1, Q3 31.82)* | *SLE patients diagnosed according to the criteria SLICC 2012, Belimumab IV* | *11 (6.5 Q1, Q3 20)* | *NR* | *Hydroxychloroquine (n = 8),  Mycophenolate mofetil (n = 6), Azathioprine (n = 4), Methotrexate (n = 2) (Sept 2017)   Azathioprine (n = 5), Methotrexate (n = 3), glucocorticoids (n = 17) (Dec 2018)* | *Initial dose: 10 mg / kg every 14 days*  *Maintenance dose: 10 mg / kg every 28 days* |
| *Riancho-Zarrabeitia, 2018* | *11* | *0 (11)* | *38.9 ± 9.6* | *SLE with articular manifestations (100%), skin (81%), hematologic (64%), kidney (27%), lung (9%) and heart (9%). Antinuclear antibodies + (n=11), native anti-DNA (27%), anti-SSA antibody (45%), anti-Ssb antibody (36%) Antiphospholipid antibody + (45%) hypocomplementemia (33%)* | *NR* | *NR* | *Antimalarials (100%) Methotrexate (over 80%) Azathioprine (27%) Anti-TNF (27%) Cyclophosphamide (18%) Leflunomide (18%) Tacrolimus and Rituximab (n = 1)* | *Treatment with Belimumab* |
| *SLE: Systemic Lupus Erythematosus; SLICC: Systemic Lupus International Collaborating Clinics; SLEDAI: Systemic lupus erythematosus disease activity index; NR: Not reported; IQR: Interquartile range* | | | | | | | | |

| TABLE 3. MAIN DEMOGRAPHIC CHARACTERISTICS, CLINICAL MANIFESTATIONS, AND INTERVENTIONS OF INCLUDED PATIENTS: PROSPECTIVE STUDIES | | | | | | | | |
| --- | --- | --- | --- | --- | --- | --- | --- | --- |
| *Author, year* | *Number of patients (n)* | *Male (Female)* | *Mean age, years (range)* | *Diagnosis* | *Mean SLE duration, years (range)* | *Mean basal SLEDAI score (E-L) (±SD)* | *Concomitant treatments, n (%)* | *Intervention* |
| *Hernández-Florez, 2015* | *8* | *NR* | *NR* | *SLE* | *NR* | *NR* | *NR* | *Treatment with Belimumab* |
| *Lorente, 2018* | *8* | *0 (8)* | *46 (25-65)* | *SLE High blood pressure (n = 6) Diabetes mellitus (n = 1) Hyperlipemia (n = 4) Ischemic heart disease (n = 4) Smoking (n = 4)* | *18 (7-27)* | *7.6 (6-12)* | *Hydroxychloroquine, 7 (87.5%) Methotrexate, 1 (12.5%) Azathioprine, 5 (62.5%) Mycophenolate mofetil, 2 (25%)* | *Belimumab iv infusion (10 mg / kg) and iv premedication (corticosteroids, acetaminophen, antihistamines) every 4 weeks.* |
| *Montserrat, 2016* | *14*  *(n=7 Belimumab)* | *NR* | *NR* | *SLE active* | *NR* | *NR* | *NR* | *Treatment with Belimumab* |
| *SLE: Systemic Lupus Erythematosus; SLICC: Systemic Lupus International Collaborating Clinics; SLEDAI: Systemic lupus erythematosus disease activity index; NR: Not reported; IQR: Interquartile range* | | | | | | | | |
